# Lessons from the Trials for the Desirable Effects of Sodium Glucose Co-Transporter 2 Inhibitors on Diabetic Cardiovascular Events and Renal Dysfunction

**DOI:** 10.3390/ijms20225668

**Published:** 2019-11-12

**Authors:** Masanori Wakisaka, Masahiro Kamouchi, Takanari Kitazono

**Affiliations:** 1 Wakisaka Naika (Wakisaka Internal Medicine Clinic), Internal medicine, Fukuoka 814-0013, Japan; 2 Department of Health Care Administration and Management, Graduate School of Medical Sciences, Kyushu University, Fukuoka 812-8582, Japan; 3 Department of Medicine and Clinical Science, Graduate School of Medical Sciences, Kyushu University, Fukuoka 812-8582, Japan

**Keywords:** sodium glucose co-transporter 2, diabetic cardiomyopathy, diabetic nephropathy, diabetic retinopathy, heart failure, pericytes, mesangial cells, fibrosis, microaneurysm, capillary leakage

## Abstract

Recent large placebo-controlled trials of sodium glucose co-transporter 2 (SGLT2) inhibitors revealed desirable effects on heart failure (HF) and renal dysfunction; however, the mechanisms underlying these effects are unknown. The characteristic changes in the early stage of diabetic cardiomyopathy (DCM) are myocardial and interstitial fibrosis, resulting in diastolic and subsequent systolic dysfunction, which leads to clinical HF. Pericytes are considered to play crucial roles in myocardial and interstitial fibrosis. In both DCM and diabetic retinopathy (DR), microaneurysm formation and a decrease in capillaries occur, triggered by pericyte loss. Furthermore, tubulointerstitial fibrosis develops in early diabetic nephropathy (DN), in which pericytes and mesangial cells are thought to play important roles. Previous reports indicate that pericytes and mesangial cells play key roles in the pathogenesis of DCM, DR and DN. SGLT2 is reported to be functionally expressed in pericytes and mesangial cells, and excessive glucose and Na^+^ entry through SGLT2 causes cellular dysfunction in a diabetic state. Since SGLT2 inhibitors can attenuate the high glucose-induced dysfunction of pericytes and mesangial cells, the desirable effects of SGLT2 inhibitors on HF and renal dysfunction might be explained by their direct actions on these cells in the heart and kidney microvasculature.

## 1. Introduction

Numbers of diabetic patients are reported to be increasing across the world. The International Diabetes Federation (IDF) estimated 451 million people had diabetes worldwide in 2017, and that the number will increase to 693 million by 2045, which leads to a large social, financial, and health system burden [1]. Among major microangiopathic complications of diabetes, nephropathy [2], cardiomyopathy [3], and retinopathy [4] have a significant impact on patient’s quality of life because they are leading causes of maintenance hemodialysis, heart failure (HF), or cardiac death and acquired visual loss, respectively. Appropriate control of blood glucose levels with insulin or sulphonylureas reduces the risk of diabetic nephropathy and retinopathy in both type 1 and type 2 diabetes [5,6].

DeFronzo et al. used phlorizin, a non-selective sodium glucose co-transporter (SGLT) inhibitor, to control blood glucose levels in diabetic rats [7]. Thereafter, phlorizin has been utilized in the treatment of experimental diabetes. Since T-1095, which inhibits renal reabsorption of glucose, was developed to treat hyperglycemia in streptozotocin-induced diabetic rats, more selective sodium glucose co-transporter 2 (SGLT2) inhibitors, which inhibit the reabsorption of glucose at the S1 segment of renal proximal tubules [8], have been developed and are currently used to treat type 2 diabetic (T2D) patients. To date, five SGLT2 inhibitors, i.e., dapagliflozin (2008: first published in a journal), canagliflozin (2010), ipragliflozin (2011), tofogliflozin (2012), empagliflozin (2012), and luseogliflozin (2013) have been prescribed for T2D patients. The reported effects of SGLT2 inhibitors include the reduction of blood glucose levels, body weight, and blood pressure, the attenuation of insulin resistance, and insulin restoration [8]. However, recent experiments or clinical trials revealed new effects of SGLT2 inhibitors.

Several recent large placebo-controlled trials of SGLT2 inhibitors evaluated the effects on cardiovascular and renal outcomes in type 2 diabetes mellitus (T2DM) patients. These trials demonstrated the desirable effects of SGLT2 inhibitors on hospitalization due to HF and renal function, with little effect on myocardial infarction and ischemic stroke. The mechanisms of these positive effects of SGLT2 inhibitors are still unknown; however, SGLT2 inhibitors seem to act mainly on microvascular disorders rather than macrovascular diseases [9,10,11,12,13], and the decrease in albuminuria was reported to be independent of glycemic control [14]. The mechanisms underlying the desirable effects of SGLT2 inhibitors seem to involve more than glycemic control.

Among diabetic complications, diabetic neuropathy, diabetic retinopathy (DR) and diabetic nephropathy (DN) are famous diabetic microangiopathies [15], and diabetic cardiomyopathy (DCM) has also been investigated since the 1970s, because DCM eventually induces HF in diabetic patients [16,17,18,19,20,21]. Myocardial and interstitial fibrosis occur in the early stage of DCM, and pericytes are considered to play crucial roles these events [21,22]. Significant correlations between DR and HF [23,24,25,26] and capillary microaneurysms derived from the loss of pericytes have been reported, and specific changes in microangiopathy were observed in both DR and DCM [23,24,25,26]. From these observations, the pathogenesis of DCM seems to be one of diabetic microangiopathy. Mesangial cells are considered to play important roles in DN [27].

Interestingly, functional expression of SGLT2 in pericytes and mesangial cells has been reported [28,29,30,31,32], and SGLT2 protein expression was revealed to increase under high-glucose conditions [28,30]. Since glucose and Na^+^ enter SGLT2 at a ratio of 1:1 [32,33], excess Na^+^ entry under high-glucose conditions might induce these cellular dysfunctions. At the same time, SGLT2 inhibitors might have direct effects on SGLT2 in pericytes and mesangial cells that evoke the preferable effects on HF and renal dysfunction.

However, the mechanisms underlying the desirable effects of SGLT2 inhibitors on HF and renal dysfunction in T2DM have not been fully elucidated. This review summarizes the outcomes of recent large placebo-controlled trials of SGLT2 inhibitors in T2DM patients and discusses their possible mechanisms.

## 2. Cardiovascular Outcomes of Large Placebo-Controlled Trials of SGLT2 Inhibitors

Among the recent studies of SGLT2 inhibitors in T2DM patients [9,10,11,12], some reported significant decreases in 3-point major adverse cardiovascular events (MACE), the occurrence of cardiovascular death [1,3] and hospitalization for HF (HHF) [9,10,11,12] in patients treated with SGLT2 inhibitors compared with the placebo-treated controls. These trials differed only in the proportions of subjects with established atherosclerosis and with multiple risk factors: 99% and 1% in EMPA-REG OUTCOME (empagliflozin), 64% and 36% in the CANVAS Program (canagliflozin) and 40% and 60% in DECLARE-TIMI 58 (dapagliflozin), respectively. Despite the diversity of atherosclerotic severity among the subjects, the hazard ratio of HHF significantly decreased in T2DM patients treated with SGLT2 inhibitors, and this result was consistent across the trials. Moreover, these favorable effects of SGLT2 inhibitors were also found in the secondary prevention of nonfatal myocardial infarction in a subanalysis of the CANVAS Program [12] and in the primary prevention of HHF in a subanalysis of DECLARE-TIMI 58 [13]. Although these trials revealed a significant decrease in the hazard ratio of 3-point MACE [9,10,11] and EMPA-REG OUTCOME reported a reduced risk of death from cardiovascular events [9], the statistical significance of these findings might disappear if death from HF was not included in the events. Additionally, a subanalysis of the CANVAS Program revealed the beneficial effects of canagliflozin on the secondary prevention of myocardial infarction, whereas other studies did not demonstrate any significant decrease in the hazard ratio of myocardial infarction [9,10,11]. Regarding cerebrovascular events, none of these studies detected any effect of SGLT2 inhibitors on the risk of acute ischemic stroke [9,10,11]. From these data, the preferable effects of SGLT2 inhibitors on cardiovascular events seem to be mainly on HF rather than on macrovascular diseases (Table 1).

## 3. Renal Outcomes of Large Placebo-Controlled Trials of SGLT2 Inhibitors

The desirable effects of SGLT2 inhibitors on the composite of renal worsening, end-stage renal disease, and renal death were consistently observed in all trials [34,35,36,37]. The trials of empagliflozin [34] or canagliflozin [35] showed a significant reduction in the progression of macroalbuminuria and lower rates of new-onset microalbuminuria and new-onset macroalbuminuria among participants with normo- or microalbuminuria at baseline. Moreover, the CREDENCE study revealed the ability of canagliflozin to significantly reduce end-stage kidney disease (ESKD) and the albumin (mg)-to-creatinine (g) ratio (UACR) and to slow the reduction in the estimated glomerular filtration rate (eGFR) at a median follow-up of only 2.62 years [36]. The CREDENCE study also performed a subgroup analysis according to eGFR at screening (30 to <45, 45 to <60, and 60 to <90 mL/min/1.73 m^2^) and to baseline UACR (> and ≤1000). Consequently, subgroups of eGFR (30 to <45, and 45 to <60 mL/min/1.73 m^2^) and UACR (>1000) showed a more pronounced reduction in the hazard ratios of the primary composite outcome ESKD, doubling of serum creatinine or death from renal or cardiovascular causes and of the renal-specific composite outcome ESKD, doubling of serum creatinine or death from renal causes [36]. The DECLARE-TIMI 58 randomized trial showed a significant reduction in the hazard ratios of the composite cardiorenal outcome of a sustained ≥40% decrease in eGFR to less than 60 mL/min/1.73 m^2^, ESKD, or death from cardiovascular or renal causes, and of the composite renal-specific outcome of a sustained ≥40% decrease in eGFR to less than 60 mL/min/1.73 m^2^, ESKD, or death from renal causes [37]. The DECLARE-TIMI 58 randomized trial also showed a significant reduction in the hazard ratios of a sustained ≥40% decrease in eGFR to less than 60 mL/min/1.73 m^2^ and ESKD [37]. The studies of SGLT2 inhibitors in T2DM patients seem to clearly reveal positive effects on urinary albumin secretion and a decrease in eGFR in DN (Table 2).

## 4. Diabetic Cardiomyopathy

Some studies since the 1970s have reported the importance of DCM in diabetic patients [10,11,12,13,14,15,16,17,18,19,20,21,22]. The Framingham Heart Study demonstrated that the occurrence of HF is 2.4- and 5.1-fold greater in male and female diabetes patients, respectively, than in age-matched control subjects [16]. Moreover, diabetes was reported to increase the risk of HF approximately 2.5-fold, independent of coronary artery disease and other comorbidities such as hypertension [20]. The characteristic changes of early stage DCM are myocardial and interstitial fibrosis and diastolic dysfunction. Subsequently, myocardial systolic dysfunction occurs, which leads to clinical HF. Myocardial and interstitial fibrosis in DCM developed independently of hypertension and coronary artery disease [21]; cardiomyocyte hypertrophy and microvascular abnormalities, such as thickening of the capillary basement membrane, have also been observed in DCM [22]. Pericytes can convert to myofibroblasts and are therefore considered to induce fibrosis in diabetic states [38]. Capillary density in the heart was lower in diabetic rats than in nondiabetic rats [39]. In DCM, the initial interstitial and perivascular fibrosis progressed more widely following replacement fibrosis accompanied by cardiomyocyte degeneration [40]. Diastolic dysfunction is a common finding in DCM; it was reported to be the first detectable functional abnormality, even in almost half of diabetic patients with good glycemic and blood pressure control and no symptoms of HF [41,42]. From these observations, pericytes in the heart seem to play important roles in cardiac fibrosis in DCM.

In the reports from the UK Prospective Diabetic Study (UKPDS) 35, significant positive associations of HbA1c levels with fatal and nonfatal myocardial infarction and stroke, microvascular endpoints, and HF were reported in T2DM patients [43]. Moreover, UKPDS 35 showed a significant 14% decrease in fatal and nonfatal myocardial infarction, a 12% decrease in fatal and nonfatal stroke, a 37% decrease in microvascular endpoints, and a 16% decrease in HF per 1% reduction in HbA1c for the 10.4-year observation period [43]. In regard to HF in T2DM patients, each 1% increase in HbA1c was reported to be associated with an 8% increased risk of HF [44]. However, one of the HF studies on the correlation between the proportion of diabetic patients who had died at the 2-year follow-up and HF according to HbA1c revealed a U-shaped association, with the lowest risk of death in patients with modest glucose control (7.1% < HbA1c ≤7.8%) [45]. In studies of the death of diabetic patients with advanced HF with reduced ejection fraction (HFrEF), deaths increased among patients with HbA1c ≥7.3% [46]. From these observations, glycemic control seems to be very important for reducing HF in diabetic patients. Meta-analyses of intensive glucose control studies, including Action to Control Cardiovascular Risk in Diabetes (ACCOD), Action in Diabetes and Vascular Disease: Preterax and Diamicron Modified Release Controlled Evaluation (ADVANCE), UKPDS, and Veterans Affairs Diabetes Trial (VADT), that used antidiabetic drugs other than SGLT2 inhibitors for glycemic control, showed a significant 15% decrease in the risk of myocardial infarction but no prevention of HF in T2DM patients in the almost five-year observation period [47]. These data imply that SGLT2 inhibitors have specific effects on HF, not glycemic control. In fact, the decrease in HbA1c attained in EMPA-REG OUTCOME, CANVAS Program, and DECLARE-TIMI 58 was very small, from 0.3% to 0.6% [9,10,11]. These facts suggest that the positive effects of SGLT2 inhibitors on HF and renal function in T2DM patients do not stem from effects on glycemic control.

## 5. Mechanisms of Heart Failure and Diabetic Retinopathy

HF is categorized into HF with preserved ejection fraction (HFpEF) and HFrEF (usually ejection fraction ≤40%) [48]. Although the effect of canagliflozin on known HFpEF was reported to be not significant, Figtree et al. reported that significant effects of canagliflozin on HFpEF and HFrEF when subjects with an unknown ejection fraction (EF) were included [49]. HFpEF is usually found in approximately 40% of diabetic and nondiabetic subjects with HF. The characteristics of HFpEF include increased left ventricular (LV) stiffness, impaired LV relaxation, decreased microvessel count and microvessel dilatory dysfunction [50,51], whereas the characteristic of HFrEF is systolic dysfunction [52,53]. The reported causes of HFrEF are the loss of myocardial mass, impaired myocardial contractility, and volume and/or pressure overload, which is thought to stem from ischemia and infarction due to coronary artery disease, uncontrolled hypertension, valvular incompetence, and microvessel disease [52,53]. Of these pathological changes, microvessel dysfunction is a mechanism of both HFpEF and HFrEF. In DCM, microaneurysms in capillaries of the heart have been reported [23]. Microaneurysms are one of the characteristic changes in DR [16]. Capillaries consist of endothelial cells and pericytes, and pericyte swelling and loss occur in the early stage of DR. Pericyte loss causes microaneurysm formation due to the vulnerability of pericyte-deficient capillary walls, which subsequently leads to capillary occlusion in the retina [24]. In fact, T2DM patients with DR were reported to have a 2.5-fold higher risk of developing HF compared to those without DR [25]. An association between the severity of DR and heart muscle perfusion was found in T2DM patients [26], and microvascular dysfunction was observed in HFpEF [54]. Capillary density was shown to be decreased in experimental studies of DCM [38,39,40]. From these longstanding studies of HF, pericytes in the heart and retina seem to play very important roles in the cause of HF due to DCM and the development of DR.

## 6. Mechanism of Diabetic Nephropathy

The number of patients with DN is increasing, and DN has become a global socioeconomic problem [55]. DN is considered to be caused by dysfunction from the glomerulus to the collecting duct (nephron) in the kidney [56]. Glomerular basement membrane thickening, mesangial expansion, and tubule-interstitial fibrosis are considered characteristic pathological changes in DN [55,56]. Interstitial tubular injury was reported to develop before glomerular dysfunction in DN, and tubulointerstitial hypoxia under diabetic conditions was suggested to be important as an early event in DN [57]. Pericytes play a crucial role in tubular interstitial fibrosis via vascular endothelial growth factor and platelet-derived growth factors [58]. In the kidney, mesangial cells are also important players in regulating glomerular functions [27]. In early DN, glomerular hyperfiltration occurs, which is explained by the glomerular hemodynamic hypothesis [59] or tubuloglomerular feedback (TGF) [60]. These mechanisms are based on the balance between glomerular afferent and efferent arteriolar tone [61]. In the diabetic state, mesangial cells lose their contractile response [62], which is thought to induce glomerular hyperfiltration [63]. Intraglomerular mesangial cells regulate glomerular circulation and filtration at the level of microvessels in glomeruli, and extraglomerular mesangial cells regulate the afferent and efferent arteriolar tone of glomeruli via vasoactive substances. In the diabetic state, intraglomerular mesangial cells are implicated in albuminuria and mesangial expansion, which occludes intraglomerular capillaries and decreases the glomerular filtration rate (GFR) [64]. Mesangial cells are also implicated in hyperfiltration and intraglomerular hypertension, which stem from cellular contractile dysfunction [65]. In the tubulointerstitial region of the kidney, the tissues are always exposed to ischemia and can be damaged, as evidenced by decreased erythropoietin production [66], which may be worsened by capillary pericyte dysfunction in the diabetic state, as seen in DR. In the diabetic state, the decreased erythropoietin production by neural crest-derived fibroblasts surrounding the renal tubules is probably due to tubulointerstitial hypoxia, which improved in T2DM patients treated with dapagliflozin [66]. The attenuation of pericytes in the diabetic kidney by SGLT2 inhibitors seems not to be a direct effect of dapagliflozin on erythropoietin-producing cells; rather, microcirculation in the interstitial region of kidney may have recovered, and serum erythropoietin levels may have increased, as reported two to four weeks after dapagliflozin administration [66]. The effects of SGLT2 inhibitors on pericytes may attenuate interstitial function in the kidney. Thus, through these mechanisms, mesangial cells and pericytes seem to play important roles in the development of DN.

## 7. SGLT in the Kidney and Heart

SGLT1 and SGLT2 are well known proteins localized in proximal tubular cells in the S3 and S1 proximal tubule segments, where they transport glucose and Na^+^ at ratios of 1:2 and 1:1, respectively [33]. SGLT1 is also expressed in the small intestine [33]. SGLT1 and SGLT2 expression in human proximal tubular cells was reported to be increased by protein kinase C (PKC) [33]. We reported functional SGLT2 expression in mesangial cells and retinal pericytes [28,29,30,31,32] and showed that SGLT2 acts as a physiological glucose sensor; moreover, pericytes and mesangial cells alter their tone via SGLT2 and Na^+^-Ca^2+^ exchangers according to glucose concentration [32]. However, SGLT2 expression in capillary endothelial cells was not observed [31]. Capillaries consist of pericytes and endothelial cells; the glomerulus consists of mesangial cells, endothelial cells and podocytes [67,68]; and capillary networks are present in the entire human body. Therefore, SGLT2 is expressed in entire organs and in tissues throughout the body, including the heart and kidney, not just in the S1 segment of the proximal tubule. SGLT1 expression was observed in normal myocardial tissue, where it was largely localized to the human cardiac myocyte sarcolemma and upregulated under ischemic and diabetic states [69]. SGLT1 has been shown to have protective effects on myocardial ischemic changes [70]. The selectivity of currently available SGLT2 inhibitors against SGLT1 seems to be sufficient to inhibit SGLT2 and have no effect on SGLT1 in the heart, since large placebo-controlled trials of SGLT2 inhibitors did not worsen ischemic myocardial infarction [9,10,11]. Moreover, arteries, including the coronary artery, supply oxygen and nutrients via capillaries. SGLT2 inhibitors may have some ability to protect against diabetes-induced arterial damage by attenuating capillary pericyte dysfunction.

## 8. Mechanisms of the Desirable Effects of SGLT2 Inhibitors

**Figure 1:**
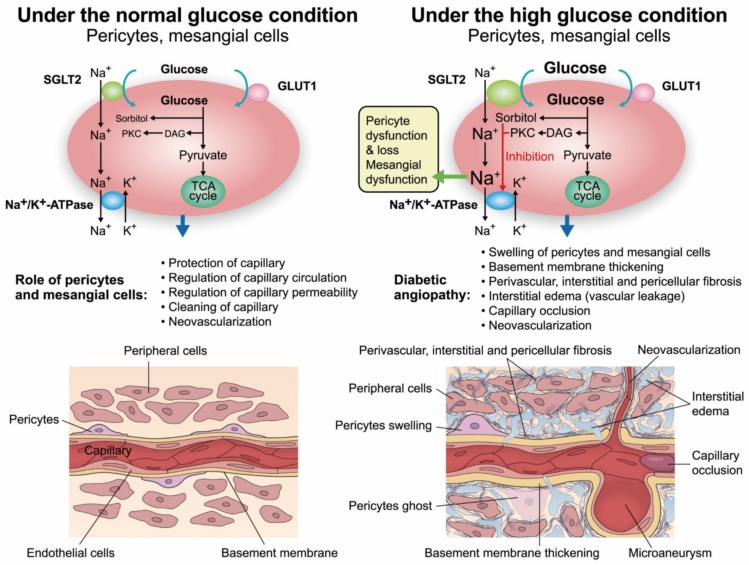
The mechanisms of capillary dysfunction in the heart and kidney and the functional expression of SGLT2 in pericytes and mesangial cells. The functional expression of SGLT2 in pericytes and mesangial cells is increased under diabetic conditions, and the uptake of both glucose and Na^+^ through SGLT2 is also increased. Intracellular sorbitol accumulation and protein kinase C (PKC) activation in response to increased intracellular glucose levels inhibit Na^+^/K^+^-ATPases. Then, intracellular Na^+^ accumulates, which leads to cell swelling and the overproduction of the extracellular matrix (upper panel). These functional changes in pericytes and mesangial cells produce perivascular and interstitial fibrosis. Myocardial fibrosis also occurs, which induces diastolic dysfunction in diabetic cardiomyopathy (DCM). These changes in pericytes and mesangial cells decrease the cellular contractile response and induce capillary structural damage, which promote pericyte loss (pericyte ghosts), microaneurysm formation, capillary occlusion and increased capillary permeability, resulting in peripheral edema (lower panel).

The results of recent large trials of SGLT2 inhibitors [9,10,11,34,35] seem to indicate the effects of SGLT2 inhibitors on capillaries in the heart and kidney, since the dysfunction of SGLT2-expressing pericytes and mesangial cells is thought to be the cause of HF and DN, as mentioned above. Mesangial cells and pericytes play crucial roles in protecting capillaries and regulating microcirculation through contractions, the phagocytosis of metabolic decomposition products around cells, neovascularization and the capillary permeability of serum substances and fluid [71,72] (Figure 1). The glucose uptake ratios of SGLT2 and GLUT1 in rat mesangial cells and bovine pericytes are almost 1:1 [25,26]. Phlorizin, an SGLT inhibitor, normalized glucose uptake by rat mesangial cells, glucose consumption by bovine pericytes, and the intracellular levels of glucose and its metabolites, such as sorbitol and fructose, in bovine pericytes under high-glucose conditions [73]. In fact, phlorizin attenuated the high glucose-induced dysfunction of rat mesangial cells and bovine pericytes, such as cellular swelling and loss, the decreased contractile response to vasoactive substances (such as angiotensin II), and the overproduction of type IV collagen [29,30,73].

We recently reported that a very low dose of canagliflozin attenuated albuminuria and pathological changes in DN in db/db mice without changing glucose levels [27]. In that experiment, SGLT2 expression in mesangial cells increased approximately five-fold under high-glucose conditions. Abnormal intracellular glucose metabolism increases intracellular sorbitol levels and activates PKC under high-glucose conditions, leading to the inhibition of Na^+^/K^+^-ATPases. Therefore, the increased SGLT2 expression leads to an increase in the simultaneous uptake of glucose and Na^+^, which induces cellular dysfunctions due to the accumulation of intracellular Na^+^ (Figure 1). However, low-dose canagliflozin was found to normalize glucose consumption and significantly decrease superoxide production and the levels of intracellular PKC, fibronectin, and transforming growth factor β1 (TGFβ1) in db/db mice. Interstitial fibrosis in the diabetic heart and kidney is an important factor in DCM and DN, in which pericytes play crucial roles [30]. Hepatic fibrosis may be triggered by stellate cells, the equivalent of pericytes in the liver. Interestingly, SGLT2 inhibitors were reported to slow the progression of fibrosis in the liver and kidney (dapagliflozin) [74] and in the heart (empagliflozin) [75] under diabetic conditions. Increased capillary permeability was observed in the heart, lung, and renal cortex and medulla in diabetic rats [76,77]. Increased capillary permeability is known to induce interstitial edema and diabetic macular edema (DME) [24,25]. We reported that half the typical dose of the SGLT2 inhibitor ipragliflozin attenuated the retinal edema and recovered the visual acuity of a diabetic woman with DME [78]. The restoration of high glucose-induced pericyte dysfunction by the SGLT2 inhibitor may have attenuated her DME.

**Figure 2:**
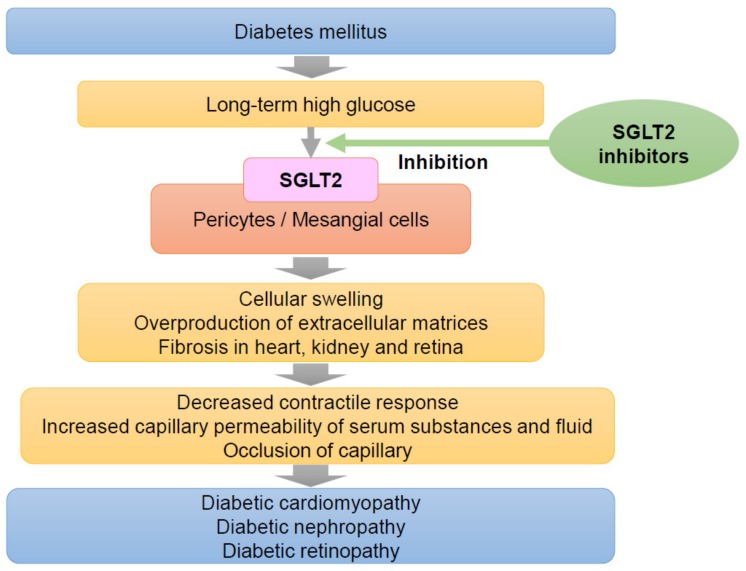
The mechanisms of SGLT2 in diabetic cardiomyopathy, nephropathy, and retinopathy. Figure 2. expression in pericytes and mesangial cells under high-glucose conditions induces capillary damage in the heart, kidney, and retina, as shown in Figure 2. The subsequent cellular dysfunction evokes DCM, diabetic nephropathy (DN), and diabetic retinopathy (DR). SGLT2 inhibitors inhibit the excess glucose and Na^+^ entry through SGLT2 into pericytes and mesangial cells, which protects against and attenuates high glucose-induced capillary damage in the heart, kidney and retina. Since capillaries are widespread in the human body, further positive effects of these inhibitors on human disease are expected.

Although the mechanisms underlying cardiorenal protection by SGLT2 inhibitors have been explained by TGF, which induces hyperfiltration in the diabetic state [60], the main cause of the positive effects of SGLT2 inhibitors is possibly independent of TGF. SGLT2 inhibitors are well-known to increase urinal Na^+^ excretions, however, decreased urinal Na^+^ excretions were reported in diabetic rats treated with an SGLT2 inhibitor compared to untreated diabetic rats [79]. Chilton suggested that the overall cardio- and reno-protective effects of SGLT2 inhibitors in T2D patients with high CV risk are most likely attributable to multiple mechanisms, including hemodynamic, metabolic, anti-inflammatory, and renal effects [80]. On the other hand, Patel et al. suggested that pleiotropic effects of SGLT2 inhibitors may be independent of glycemic control for cardiovascular diseases, HF and chronic kidney disease with or without T2D [81]. Indeed, a very low dose of an SGLT2 inhibitor attenuated diabetic glomerular pathological changes and urinary albumin excretion, as mentioned above [30]. The very low dose of canagliflozin in our experiment with db/db mice seemed to produce an effective concentration in plasma or local renal tissue, as evidenced by SGLT2 inhibition in mesangial cells and pericytes without any effect on SGLT2 in the proximal tubules, since there was no effect on blood glucose levels. The IC_50_ of canagliflozin to inhibit alpha-methyl-D-glucopyranoside transport by human SGLT1 and SGLT2 is reported to be 663 and 4.2 nM, respectively [82], and the excretion of canagliflozin in urine is <1% of the administered dose [83]. Moreover, phlorizin normalized the high glucose-induced reduction in the contractile response of mesangial cells, as mentioned in the mechanism of DN section. The desirable effects of SGLT2 inhibitors on HF and renal dysfunction, as microvascular disorders, could be explained by their actions on mesangial cells and pericytes, whereby they attenuate high glucose-induced fibrosis and the capillary permeability of serum substances and fluids (Figure 2).

## 9. Treatments for HF and DN Other Than Glucose-Lowering Drugs

The American Heart Association stated that angiotensin converting enzyme inhibitors (ACEIs) or angiotensin receptor inhibitors (ARIs) can be useful for preventing HF in diabetic patients with hypertension at high risk for developing HF but with no functional or structural cardiac disorders and good glycemic control (HbA1c <7.0 and without hypoglycemia) [84]. In DN, inhibition of the renin-angiotensin system (RAS) by ACEIs or ARIs was reported to reduce the incidence of microalbuminuria in T2DM patients [85]. RAS inhibitors have also been reported to reduce the risk of DR [86]. These results suggest that RAS inhibition might have positive effects in the early stages of DN and DR in T2DM patients. Interestingly, ACEIs were reported to decrease proximal tubular SGLT2 protein levels compared to controls in diabetic rats [87]. Captopril (an ACEI) at a concentration of 10^-4^ M was reported to suppress Na^+^-dependent glucose uptake and alfa-methyl glucoside uptake; these substances enter cells through SGLT2, attenuate the intracellular levels of glucose and glucose metabolites, such as sorbitol and fructose, under high-glucose conditions, and normalize the high glucose-induced swelling of bovine retinal pericytes [88]. Based on these results, RAS inhibitors might act as weak SGLT2 inhibitors and might reduce the risk of diabetic complications.

Pioglitazone (a thiazolidinedione) was reported to reduce cardiovascular risk [89]. The effects of pioglitazone on cardiovascular outcomes were revealed to be significant: there was an 18% reduction in the hazard ratio of ischemic stroke and a 26% reduction in myocardial infarctions in the combined data of the PROactive study [90] and IRIS [91]. Regarding macrovascular complications, pioglitazone seems to have more pronounced and preferable effects than SGLT2 inhibitors [9,10,11]. However, since pioglitazone has some effects on Na^+^ retention [92], it was reported to occasionally induce HF [93]. Regarding DN, urinary albumin excretion decreased in T2DM patients treated with pioglitazone [94,95]; however, a greater decline in eGFR was shown in T2DM patients treated with pioglitazone than in those treated with placebo [96]. From these reports, pioglitazone seems to be more effective in macrovascular diseases than in DN. Pioglitazone is a specific ligand of peroxisome proliferator-activated receptor γ (PPARγ), a nuclear receptor that plays an important role in regulating cell differentiation at the transcriptional level [97]. PPARγ in smooth muscle cells, macrophages, and endothelial cells has been reported to play important roles in the pathogenesis of atherosclerosis [98,99,100]. The inhibitory effects of pioglitazone on the progression of atherosclerosis in cellular models have also been reported [100,101]. At the microvascular level, pericytes and mesangial cells were reported to functionally express PPARγ1 and PPARγ2 [102,103], respectively. PPARγ1 in rat mesangial cells was downregulated by PKC activation, and the downregulation of PPARγ1 was reported to prompt the loss of the contractile response to angiotensin II, as observed in DN, which was recovered by pioglitazone [104]. Interestingly, troglitazone (another thiazolidinedione) was shown to increase glucose uptake by rat mesangial cells through GLUT1 under normal and high-glucose conditions; however, intracellular glucose content did not increase under normal glucose conditions, and intracellular sorbitol levels were significantly decreased under high-glucose conditions [105]. Pioglitazone also increased glucose uptake by astrocytes through GLUT1 and protected these cells against hypoglycemia-induced death [106]. Moreover, troglitazone significantly decreased the intracellular redox potential under normal and high-glucose conditions [105]. The decreases in intracellular glucose and ROS by thiazolidinediones indicate the normalization of abnormal glucose metabolism under high-glucose conditions in the presence of SGLT2 inhibitors [27,73]. The differences between thiazolidinediones and SGLT2 inhibitors include their effects on sodium and glucose entry into cells, namely, SGLT2 inhibitors increase Na^+^ excretion through urine and decrease Na^+^ and glucose entry into pericytes and mesangial cells, and thiazolidinediones occasionally increase Na^+^ retention by the kidney and increase glucose uptake by mesangial cells without increasing intracellular glucose metabolites and ROS. DeFronzo et al. suggested that combination therapy with pioglitazone and SGLT2 inhibitors might reduce further cardiovascular events in high-risk T2DM patients [107].

## 10. Conclusions

The results of large placebo-controlled trials on SGLT2 inhibitors regarding cardiovascular and renal outcomes revealed the positive effects of these inhibitors on HF and renal function [9,10,11,14,15]. These findings suggest that SGLT2 inhibitors are mainly effective against microvascular diseases rather than macrovascular diseases. Further analyses are being conducted to validate these findings. Pericytes in capillaries and mesangial cells seem to play key roles in the development of DMC and DN, including in perivascular and cellular fibrosis, microaneurysm formation in capillaries and capillary occlusion, which are diabetic-specific microangiopathies. Because SGLT2 is functionally expressed in pericytes and mesangial cells and excess glucose and Na^+^ enters through SGLT2 in diabetic states induce these cellular damages, the preferable effects of SGLT2 inhibitors on HF and renal dysfunctions are probably thought to be independent of the glucose control, and to be mainly derived from direct actions of SGLT2 inhibitors on mesangial cells and pericytes.

## Figures and Tables

**Table 1:** Effects of SGLT2 inhibitors on cardiovascular events.

	Empagliflozin	Canagliflozin	Dapagliflozin
3point MACE	Sinificantly desirerable	N.S.	N.S.
Primary 3poin MACE	N.D.	Sinificantly desirerable	N.D.
CV death	Sinificantly desirerable	N.S.	N.S.
HHF	Sinificantly desirerable	Sinificantly desirerable	Sinificantly desirerable
non-fatal MI	N.S.	N.S.	N.S.
non-fatal stroke	N.S.	N.S.	N.S.
Primary HHF	N.D.	N.D.	Sinificantly desirerable
Pre-MI history	N.D.	Sinificantly desirerable	N.S.
Reference No.	9	10, 12	11, 13

N.S.: not significantN.D.: not determined; HHF: hospitalization for heart failure; MI: myocardial infarction.

**Table 2:** Effects of SGLT2 inhibitors on renaldysfunction.

	Empagliflozin	Canagliflozin	Dapagliflozin
Composite of renal worsening			
end-stage renal disease, and renal death	Sinificantlydesirerable	Sinificantlydesirerable	Sinificantlydesirerable
Progression of macroalbuminuria	Sinificantly desirerable	Sinificantly desirerable	N.A.
new onset of microalbuminuria	Sinificantly desirerable	Sinificantly desirerable	N.A.
new onset of microalbuminuria	Sinificantly desirerable	Sinificantly desirerable	N.A.
occurrence of ESKD	Sinificantly desirerable	Sinificantly desirerable	N.A.
reduction of UACR	N.A.	Sinificantly desirerable	Sinificantly desirerable
reduction of eGFR	Sinificantlydesirerable	Sinificantlydesirerable	Sinificantly desirerable
Reference	34	35, 36	37

N.A.: not available; ESKD: endsatgekidney disease; UACR: albumin(mg)-to-creatinine (g) ratio; eGFR: estimatedglomerular filtration rate.
